# Ruptured Sinus of Valsalva Aneurysm with Resultant Myocardial Pouch Formation in the Fetal Heart—A Diagnostic Challenge

**DOI:** 10.3390/jcdd11010023

**Published:** 2024-01-14

**Authors:** Hugh Bigg, Elijah Bolin, Dala Zakaria, Renee Bornemeier

**Affiliations:** 1 Division of Pediatric Cardiology, University of Utah, Primary Children’s Hospital, #100 Mario Capecchi Dr., Salt Lake City, UT 84113, USA; 2 Section of Cardiology, Department of Pediatrics, University of Arkansas for Medical Sciences College of Medicine, Arkansas Children’s Hospital, #1 Children’s Way, Slot 512-3, Little Rock, AR 72202, USA

**Keywords:** sinus of Valsalva, aneurysm, double-inlet left ventricle

## Abstract

Sinus of Valsalva aneurysms (SVAs) are infrequently seen in the pediatric population. When these aneurysms rupture, a significant hemodynamic burden is placed on the heart and increases the likelihood of cardiac failure. Here, we report a case of a ruptured SVA into the ventricular myocardium in a fetus with a form of double-inlet left ventricle. To the best of our knowledge, this has not previously been described.

## 1. Introduction

It is rare to identify findings in utero consistent with those of aortic insufficiency. However, when a reversal of flow is seen in the ascending aorta or a to–fro flow is found in the region of the aortic root, diagnostic consideration for some of the rarest of congenital cardiac anomalies must be given [1,2,3,4,5]. A left ventricular to aortic tunnel or the absence of aortic valve leaflets would be the two most likely malformations accounting for these types of findings. These anomalies result in an aortic insufficiency-type burden within the fetal heart, which may create such a hemodynamic burden that fetal hydrops and even fetal demise can occur [1,2,3]. We present a very unusual cause for such a finding: a ruptured sinus of Valsalva aneurysm into the ventricular myocardium of a fetus with a single left ventricle.

Sinus of Valsalva aneurysms (SVA) are rare anomalies most commonly observed in adults [6,7,8]. The incidence, in the general population, has been reported to be 0.09% [9]. Rupture of these aneurysms can acutely increase the cardiac afterload and impose a significant increased hemodynamic demand on myocardial function. Heart failure can result. Children account for fewer than 3% of all cases [8], with only a handful of neonatal cases reported in the literature [10,11,12,13,14,15,16,17,18,19]. Identification of a sinus of Valsalva aneurysm, unruptured or ruptured, in the fetus is extremely rare. Only two such case reports have been found: one unruptured and prenatally diagnosed; the other having a prenatal diagnosis of hypoplastic left heart syndrome with the additional diagnosis of a ruptured SVA conducted on the initial postnatal echo [20,21]. Given the uniqueness of such a defect, we report our case to the overall body of knowledge for the consideration of such a rare lesion.

## 2. Case Description

A 19-year-old woman was referred by her primary obstetrician to our maternal–fetal medicine specialists after a level two ultrasound revealed multiple extracardiac anomalies. A 33-week anatomy scan showed an estimated fetal weight at the 17th percentile for gestation, a two-vessel cord with absent left kidney and pelvic right kidney, short left forearm, and a clenched right hand. A congenital heart defect was noted with what was reported to be a large ventricular septal defect (VSD). Additional concerns were raised for a cardiac tumor, with an unusual “cardiac mass” also observed. Non-invasive prenatal testing (NIPT) was performed and was low risk.

**Figure 1:**
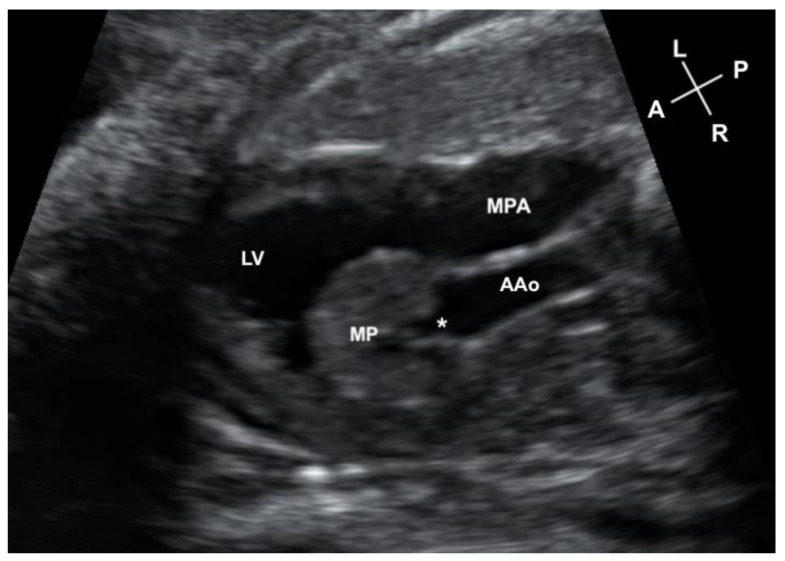
Axial image on the fetal scan of the myocardial pouch (MP) protruding into the left ventricular cavity (LV) and the connection of the pouch (*) to the ascending aorta (AAo). The main pulmonary artery (MPA) is seen to the left of the ascending aorta.

**Figure 2:**
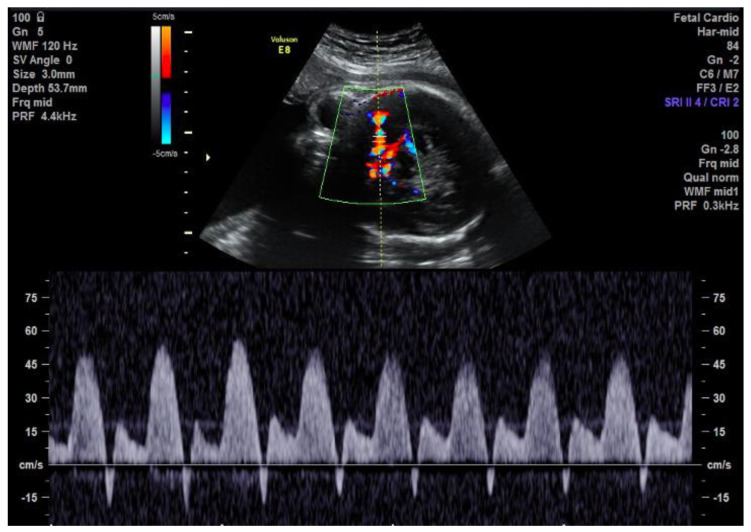
Brief early diastolic reversal of flow in the MCA seen on the spectral Doppler due to the reversal of flow in the ascending aorta.

An urgent fetal echocardiogram and cardiology consultation were requested and performed the same day. The cardio-thoracic area ratio was increased at 42%. There was atrio-ventricular synchrony with a fetal heart rate of 142 beats per minute. The fetal echocardiogram demonstrated double-inlet left ventricle (DILV) with left atrioventricular valve atresia. The left ventricle was dilated with preserved systolic function. There was a superior, leftward-positioned infundibulum giving rise to a well-formed pulmonary artery. A moderately sized VSD connected the left ventricular cavity and the sub-pulmonary chamber. The aorta’s connection to the base of the heart was not well delineated, but the aorta was positioned to the right of the pulmonary artery. Aortic valve leaflets were difficult to discern on the fetal scan, and the arch was hypoplastic. Flow consistent with severe aortic insufficiency was observed, with diastolic flow reversal in the ascending aorta. This reversed flow entered into a contracting myocardial “pouch” which had invaginated into the superior aspect of the left ventricular cavity (Figure 1). The pouch was not connected to the left ventricular cavity or the sub-pulmonary chamber/infundibulum. The “pouch” filled in diastole and in systole, contracted, ejecting into the ascending aorta. There was diastolic flow reversal in the hypoplastic transverse aortic arch and innominate artery by color and spectral Doppler. The middle cerebral artery demonstrated brief, early diastolic flow reversal (Figure 2). Aortic valve leaflets could not be defined; therefore, it was not clear where the aortic valve annulus may be, if the reversal of flow was caused by the absence of aortic valve leaflets, or whether there was a tunnel allowing the aortic root to communicate with the myocardial “pouch”. The family was counseled regarding options for post-natal management. Concerns for how well the fetus could tolerate the ongoing aortic insufficiency throughout the remainder of gestation, and how this may impact what options could be considered postnatally, were discussed. There was no evidence of hydrops fetalis throughout our surveillance of the fetus.

The child was born by a repeat low transverse Cesarean section at 39 weeks 2/7 days gestation weighing 2500 gm. Apgar scores were 5 and 8 at 1 min and 5 min, respectively. The infant was noted to have dysmorphic features with posteriorly rotated and low-set ears, left ear helix fusion, and absent left radius and right first metacarpal bones. Renal ultrasound confirmed an absent left kidney and a pelvic right kidney. Genetic testing was performed, which returned a variant of unknown significance at 2p16.3, including the NRXN1 gene and two benign copy number variants. Although reports exist with patients having deletions at 2p16.3 involving the NRXN1 region and cardiac defects, the frequency of these findings across all reports has been variable [22], and it is inconsistent to draw specific conclusions.

The infant initially required continuous positive airway pressure at 8 cm H_2_O of 21% FiO_2_ to sustain arterial oxygen saturations greater than 75%. There was no difference between pre- and post-ductal arterial oxygen saturations. Prostaglandin was initiated at 0.05 mcg/kg/min through an umbilical venous line.

**Figure 3:**
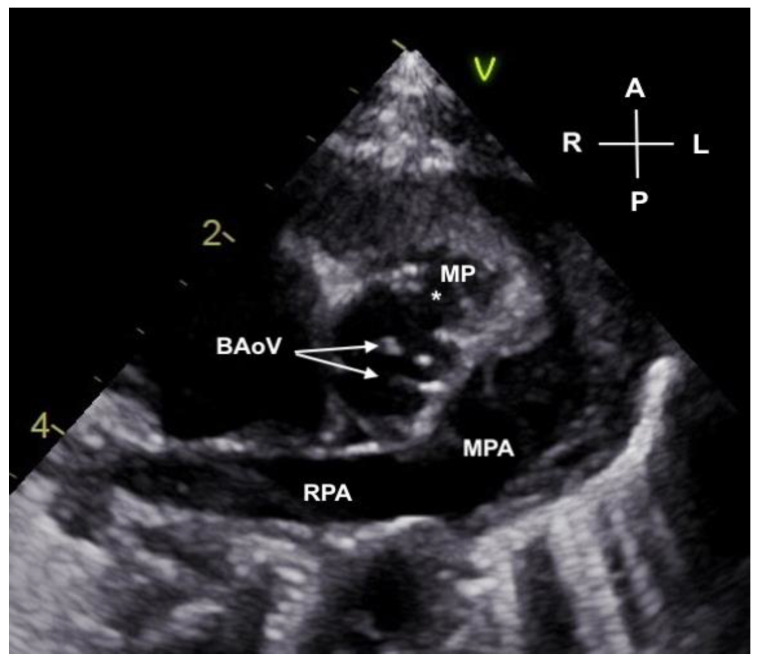
Parasternal short axis view of the bicuspid aortic valve (BAoV) with valve leaflets noted by arrows. A deficiency in the anterior sinus of Valsalva (*) has “mined” a cavity into the surrounding myocardium creating a pouch (MP).

**Figure 4:**
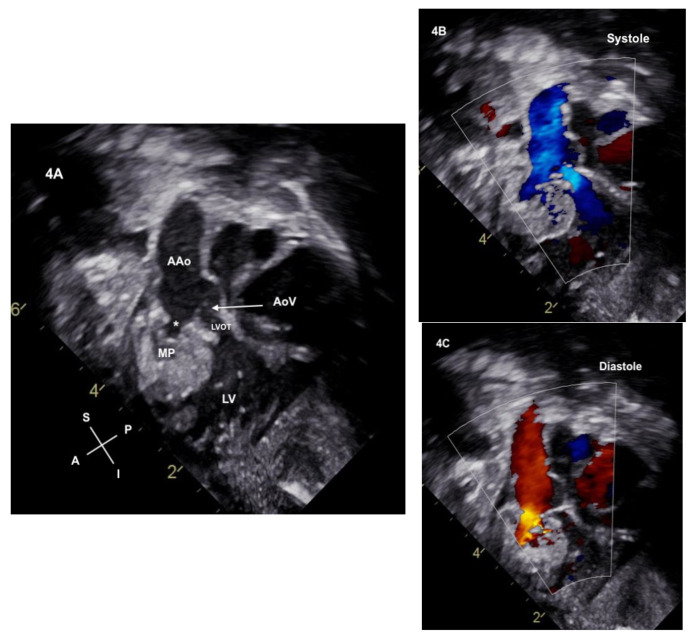
(**A**) Neonatal echocardiogram from the subcostal sagittal plane demonstrates the aortic valve (AoV) leaflets (denoted by the arrow) which were not seen on the fetal scans. The aortic valve annulus is hypoplastic with a narrowed left ventricular outflow tract (LVOT). The ruptured sinus connection (*) to the myocardial pouch (MP) is noted. (**B**) Color is now applied and antegrade flow is noted through the LVOT and into to the ascending aorta. Antegrade flow is also seen from the myocardial pouch into the ascending aorta. (**C**) Color in diastole demonstrates the reversal of flow from the ascending aorta into the myocardial pouch through the ruptured sinus of Valsalva. Notably, no aortic valve insufficiency is seen when the valve leaflets are closed, and no diastolic flow reversal is noted in the LVOT.

Transthoracic echocardiography confirmed DILV with left atrioventricular valve atresia. A hypoplastic aorta, originating from the left ventricle, posterior and rightward to the pulmonary artery, was noted. Surprisingly, a bicuspid aortic valve, with fusion of the right and left cusps, was present, and the leaflets coapted without insufficiency. However, the aortic annulus was hypoplastic. Upon further investigation of the aortic root, a deficiency in the wall of the anterior sinus of Valsalva was identified as the cause of the flow reversal and the “insufficiency” noted prenatally (Figure 3). This flow from the ruptured sinus of Valsalva had “mined” a cavity into the ventricular myocardium, forming a contractile pouch with to–fro flow into and out of the pouch (Figure 4A–C). Coronary arteries arose from their respective right and left sinuses from the anterior fused cusps of the bicuspid aortic valve.

The family elected to proceed with hybrid palliation, which was completed on the ninth day of life. The hybrid was performed in the cardiac catheterization lab via a median sternotomy. Bilateral pulmonary artery bands were placed and, with the chest open, a sheath was inserted directly into the main pulmonary artery and two palmar stents were placed into the ductus arteriosus. The second stent was placed because there was concern that a small portion of the distal ductus was not fully covered by the first stent. The atrial level shunt was unrestrictive and left-to-right, so a septostomy was not performed. Diastolic blood pressures were normal; therefore, no interventions on the ruptured sinus or myocardial pouch were performed. Consideration for intervention on the pouch was to be determined over time based on the continued monitoring of growth or impingement on adjacent structures.

**Figure 5:**
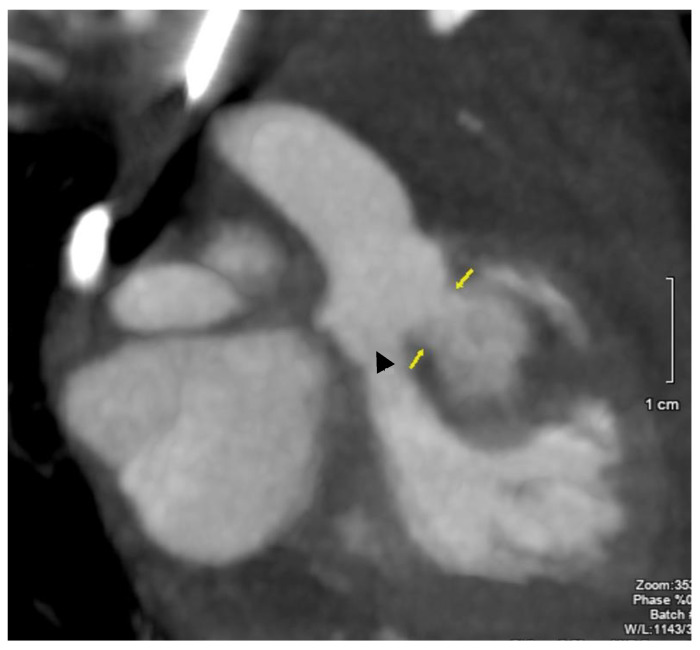
Coronal visualization by CT of the aortic root. Boundaries of the ruptured right sinus of Valsalva aneurysm into the myocardial pouch is noted by the arrows. The triangle (▲) points to the location of the aortic valve leaflets.

**Figure 6:**
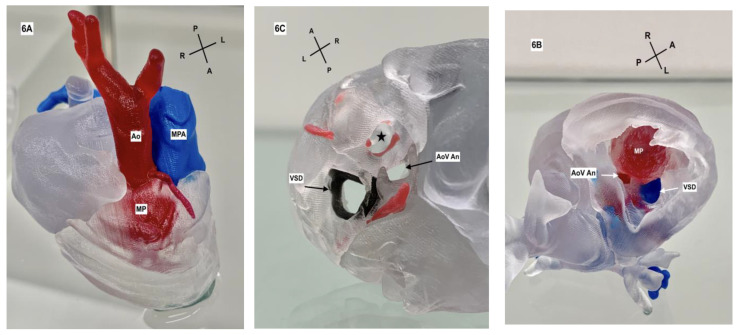
(**A**) Three-dimensional model of the heart, looking down from a superior view. The aorta (Ao) can be seen arising to the right of the main pulmonary artery (MPA). Below the translucent myocardium in this model, the myocardial pouch can be noted. (**B**) Three-dimensional model of the heart with the diaphragmatic surface of the LV myocardium removed. Looking from inferior to superior, the hypoplastic aortic valve annulus can be seen (AoV An) along with the ventricular septal defect (VSD) with the pulmonary artery seen in blue. The myocardial pouch (MP) has invaginated into the superior aspect of the left ventricular myocardium. (**C**) A second 3D model from a superior view demonstrating the VSD opening (black ring) and the AoV An. The star is positioned in the defect of the sinus of Valsalva and is the opening into the myocardial pouch.

The infant was extubated one week after the procedure, but could not be weaned off supplemental oxygen and fed poorly. At 2 months of life, a deterioration in systolic function developed. The etiology for the decline in function could not be identified, and the family elected to withdraw care. Cardiology was not afforded the opportunity of an autopsy, but a computed tomography (CT) scan had been performed which demonstrated the anatomy (Figure 5), and a 3D model was constructed from the CT dataset acquired on the infant’s third day of life (Figure 6A–C).

## 3. Discussion

As previously stated, findings consistent with aortic valve insufficiency in the fetal heart are unusual. When present, the etiology typically includes very rare forms of congenital heart disease, including a left ventricular to aortic tunnel or absence of the aortic valve leaflets. It is unusual to see neonates with these types of lesions, due to their overall rarity and the potential for intrauterine demise from such a hemodynamic burden placed on the heart.

SVAs are typically found in older populations and are not often seen in infancy. Rupture of these aneurysms usually occurs into a ventricular cavity, or occasionally the atrium. SVAs usually arise from the base of one of the aortic sinuses [14] between the tunica media and intima [23], and most commonly involve the right sinus (70–90%) [24], with the second most common sinus being the non-coronary sinus. The sinus least often associated with a sinus of Valsalva aneurysm and rupture is the left aortic sinus. SVAs can occur in the presence of genetic disorders such as connective tissue anomalies, Noonan’s syndrome [25,26], and trisomy syndromes. They can also be associated with congenital cardiac disease such as conoventricular or outlet ventricular septal defects, bicuspid aortic valves, aortic insufficiency, and coronary anomalies [24], and have been reported in myopathies such as LV non-compaction [8,27]. Ruptured SVAs may have an infectious etiology [18] or can result from trauma [28,29].

The timing of diagnosis depends on clinical suspicion and symptoms that are related to the presence or absence of rupture. Up to 66% of SVAs are ruptured at the time of diagnosis [24], resulting in a large left-to-right shunt into the right ventricle (70–90%) or right atrium (10–20%). Rupture into the left ventricle (<5% of cases) and interventricular septum (approximately 2% of cases) are much less common [30,31,32]. While rupture into the interventricular septum can be asymptomatic, sub pulmonary outflow tract obstruction can result [33], hastening signs and symptoms. The overall mean age at diagnosis is in the third decade of life [12], with a 3–4:1 male predominance. Autopsy studies have estimated the incidence in the general population to be 0.09%, although it is five times more common in people of Asian ancestry compared with those of European heritage. Autopsy studies likely underestimate the true incidence, as reports regarding patients undergoing open heart surgery have suggested an incidence of 0.15% to 3.5% [24,34].

Ruptured SVAs in pediatric patients account for 0.1% to 3.5% of congenital heart disease, and 3% of all cases [23]. Neonatal diagnosis is not only exceptionally rare, but also difficult to make, with symptoms varying from “severe heart failure” [12] to an isolated murmur [10] (systolic [8], diastolic, and/or continuous [35]). Older children and adults may report dyspnea, palpitations, dizziness, or syncope. Pediatric patients, like adults, can demonstrate the full spectrum of presentations. In an unruptured state, some may be asymptomatic or be incidentally identified at autopsy. Once ruptured, the diagnosis is typically made due to changes in exam findings or the development of symptoms and require surgical intervention [10]. 

With the rarity of a ruptured SVA described in fetal life, our knowledge of the typical “expected” findings are limited but could potentially be inferred from lesions with similar hemodynamic effects, such as LV-Ao tunnels and absent aortic valve leaflets. In this case, the postnatal echocardiographic findings of the hypoplastic bicuspid aortic valve without regurgitation and the LVOT with no diastolic reversal present were key in removing these latter two defects from the differential diagnosis.

## 4. Conclusions

In our case, with such a rare defect and the difficulty in being able to delineate aortic valve leaflets on the fetal echocardiogram, our differential diagnosis did not include rupture sinus of Valsalva aneurysm. With absent aortic valve leaflets or left ventricular to aortic tunnels, the ventricular diastolic burden is high. There is a likelihood of developing ventricular dysfunction with progression to fetal hydrops and even intrauterine demise. Although cardiac compromise did not occur in this fetus, it is plausible to assume that had the rupture of this sinus been into the atrium or ventricle, the fetus would also have been at risk for myocardial dysfunction and even fetal demise.

To the best of our knowledge, this is the first report of the prenatal diagnosis of a ruptured sinus of Valsalva aneurysm. Postnatal echocardiography and advanced imaging enabled improved visualization of the aortic root and an accurate diagnosis of the congenital cardiac anomaly present. It is possible that in the setting of our patient, the hemodynamic burden imposed upon the fetal heart was minimized due to the small size and pulsatile nature of the “cardiac pouch”. The fact that the cardiac pouch connected only to the aortic root may have lessened the diastolic burden on the ventricle, compromising the function. The incidence of sinus of Valsalva aneurysms and rupture in the fetus has not been reported, and must be extremely rare; however, with the findings in this case, this anomaly may need to be included in the differential when “aortic insufficiency-type” findings are identified in utero. With reports of these types of anomalies and further advancements in imaging capabilities in the fetal heart (fetal cardiac MRI), opportunities to provide families with more specific diagnosis prior to birth may be possible. This may also allow for a more specific plan for surgical intervention to be constructed prenatally.

## Data Availability

No new data were created or analyzed in this study. Data sharing is not applicable to this article.
